# Supplementary material for: Evidence of Biparental Mitochondrial Inheritance from Self-Fertile Crosses between Closely Related Species of Ceratocystis
Source: J Fungi (Basel). 2023 Jun 19;9(6):686. doi: 10.3390/jof9060686 (PMC10300817; doi:10.3390/jof9060686)
Supplement: Supplementary file 1 [file jof-09-00686-s001.zip › jof-2393856-supplementary.pdf]

a)

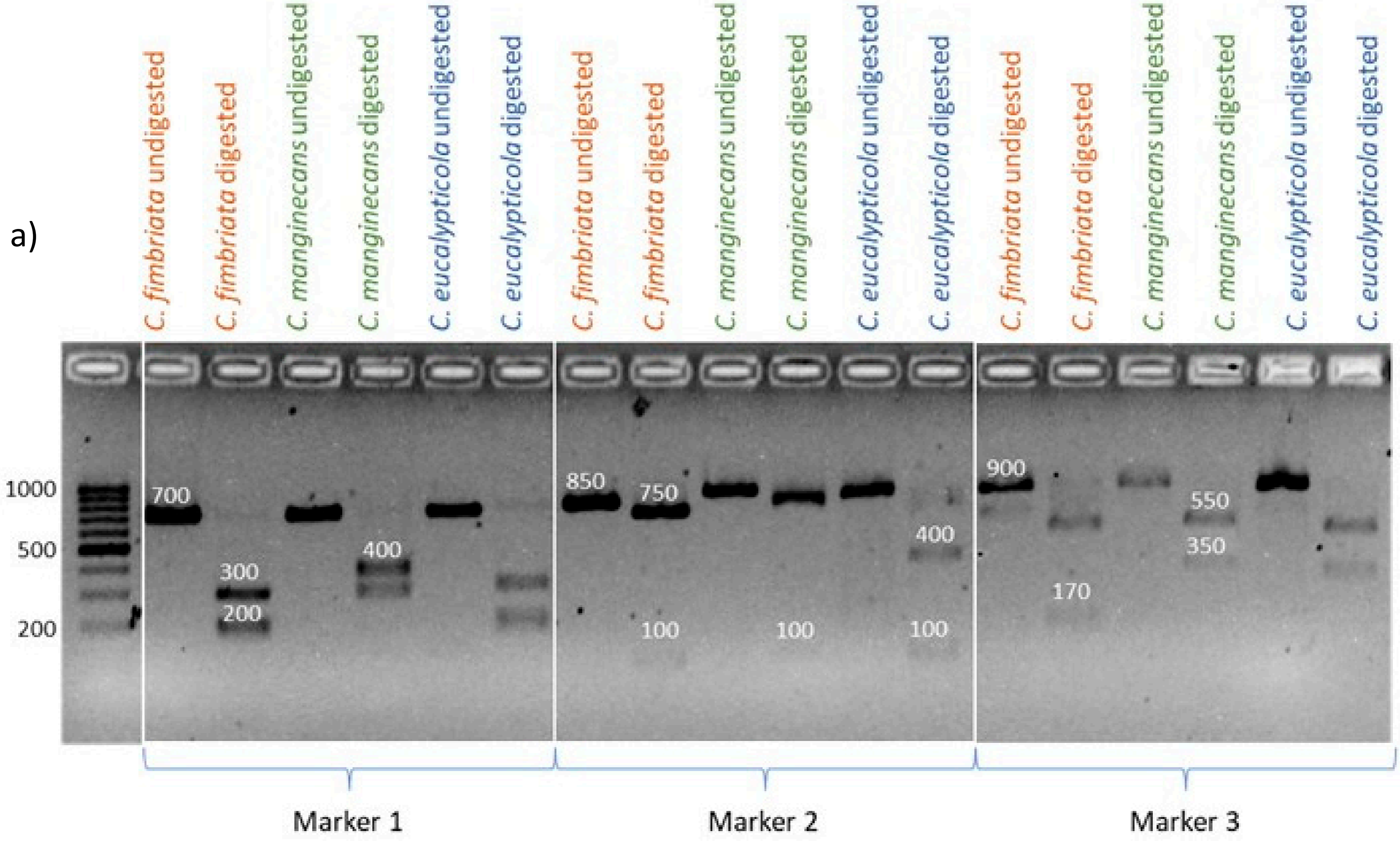
C. fimbriata undigested
C. fimbriata digested
C. manginecans undigested
C. manginecans digested
C. eucalypticola undigested
C. eucalypticola digested
C. fimbriata undigested
C. fimbriata digested
C. manginecans undigested
C. manginecans digested
C. eucalypticola undigested
C. eucalypticola digested
C. fimbriata undigested
C. fimbriata digested
C. manginecans undigested
C. manginecans digested
C. eucalypticola undigested
C. eucalypticola digested
1000
500
200
700
300
200
400
850
750
100
100
400
100
900
550
350
170
Marker 1
Marker 2
Marker 3

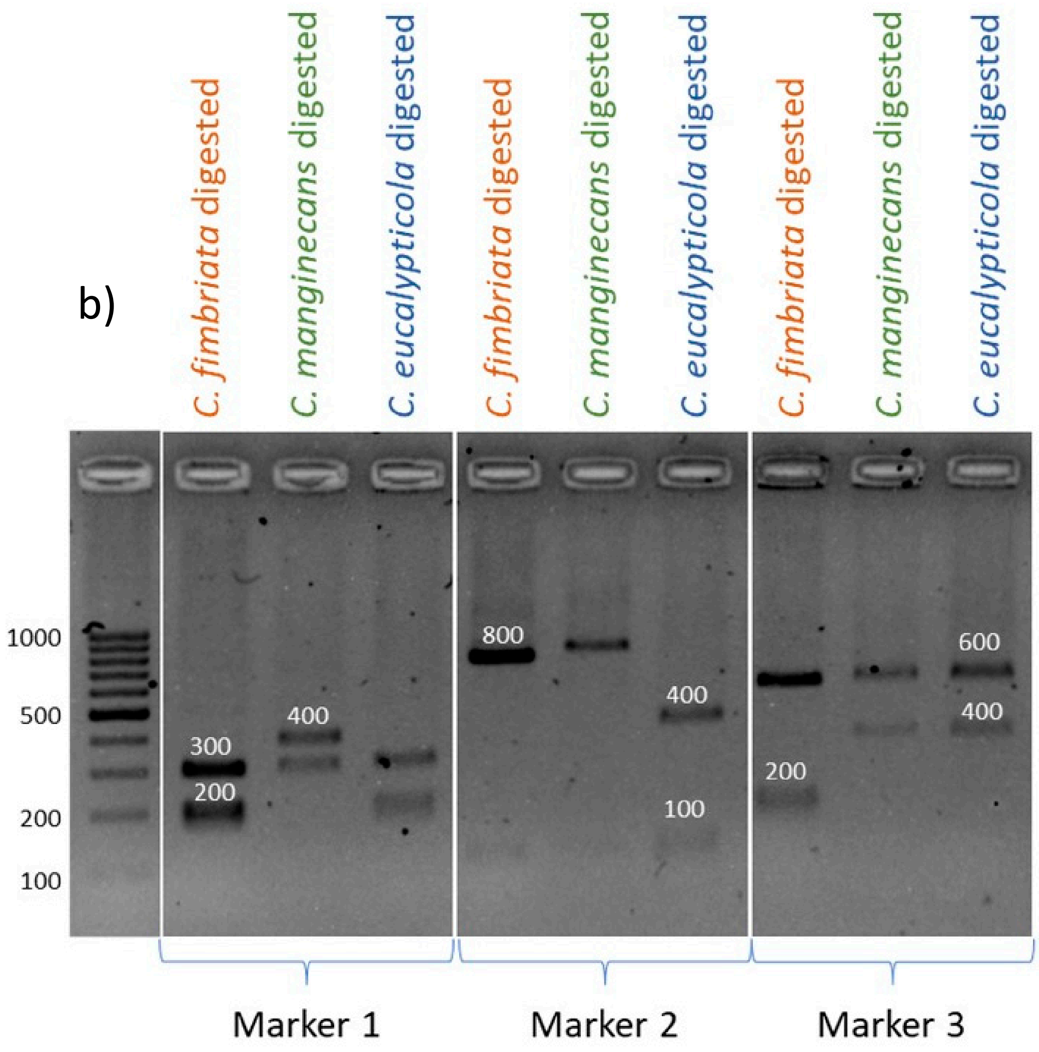

Figure S1. Amplicons and digested amplicons produced by the RFLP analysis of the three nuclear markers. Two agarose gels showing the three markers amplified from the three parent *Ceratocystis* species. Gel a) shows the obtained PCR amplification and restriction profiles for each nuclear marker set across all three species. Gel b) compares the actual restriction profiles for all three nuclear markers across all three species. Marker 1 produces an amplicon of nearly 700 bp in all three species. In *C. fimbriata* and *C. eucalypticola* , the HindIII and PstI cut sites produced bands of two sizes; 300 and 200 bp estimated respectively. The small band of 35 bp cannot be seen and two bands of 170 and 190 bp are too close in size to differentiate. There is one fewer PstI cut sight in *C. manginecans*, and so the 300 bp band is common among all three species but in *C. manginecans* the two bands around 200 bp are not separated and so make one 400 bp band. Marker 2 produces an amplicon of nearly 850 bp. There is a single HindIII cut site in *C. fimbriata* and *C. manginecans* which produces two bands of sizes 100 and about 750 bp. However, there are more HindIII and PstI cut sites present in *C. eucalypticola* , resulting in a common band of size 100 bp in all three isolates but the 750 bp band is further fragmented to produce 3 similarly sized bands that are around 100 bp in size and one 400 bp band. Marker 3 produces an amplicon of around 900 bp in each of the species. A double digestion of these amplicons with HindIII and PstI produces two bands of 550 and 350 respectively in *C. manginecans* and *C. eucalypticola* . An extra PstI cut site in *C. fimbriata* results in the 350 bp band to be cut in half again, producing two more bands of 180 and 160 bp, which are too close in size to differentiate on the agarose gel.

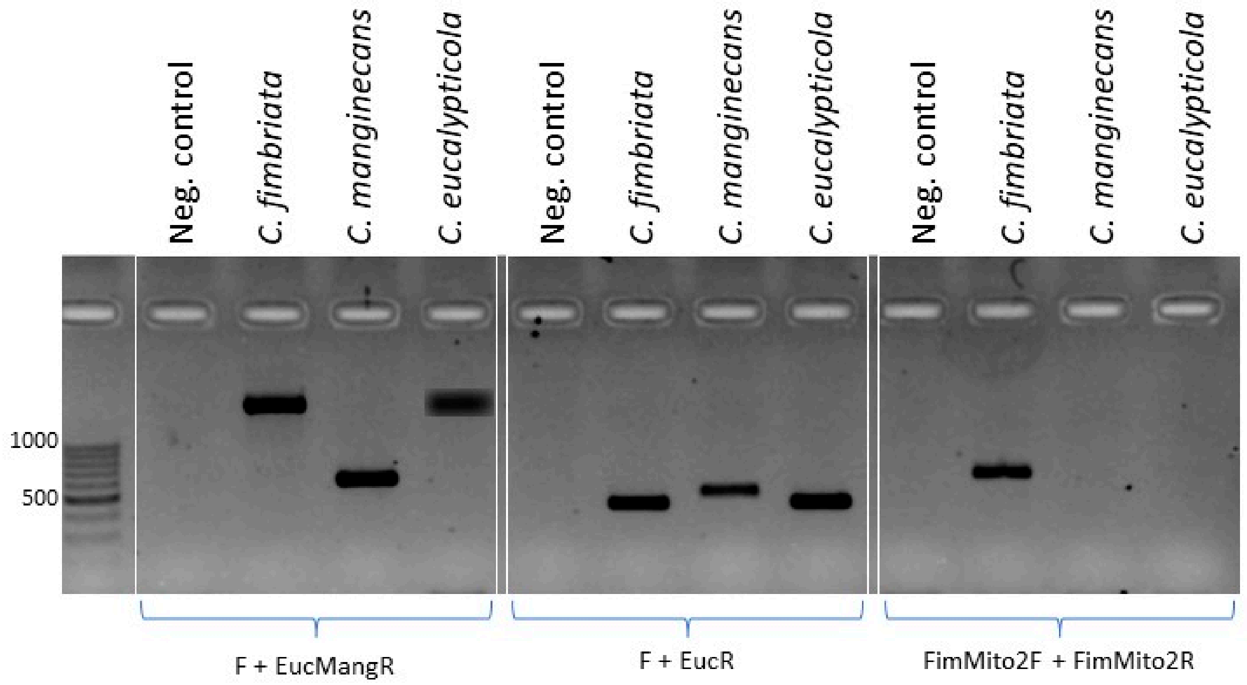

Figure S2 Amplicons produced by mitochondrial markers. Agarose gel showing amplification products of the diagnostic regions of the mitochondrial genomes of *C. fimbriata*, *C. manginecans* and *C. eucalypticola*. Primer set 1 produces an amplicon of around 1890 bp in *C. fimbriata* and *C. eucalypticola*, and an amplicon of around 560 bp in *C. manginecans*. Primer set 2 produces an amplicon of nearly 400 bp in both *C. fimbriata* and *C. eucalypticola*. An approximately 500 bp fragment was produced in *C. manginecans* although this fragment was not predicted in the initial *in silico* analysis. Primer set 3 only produces an amplicon in isolates containing mitochondrial DNA of *C. fimbriata*.

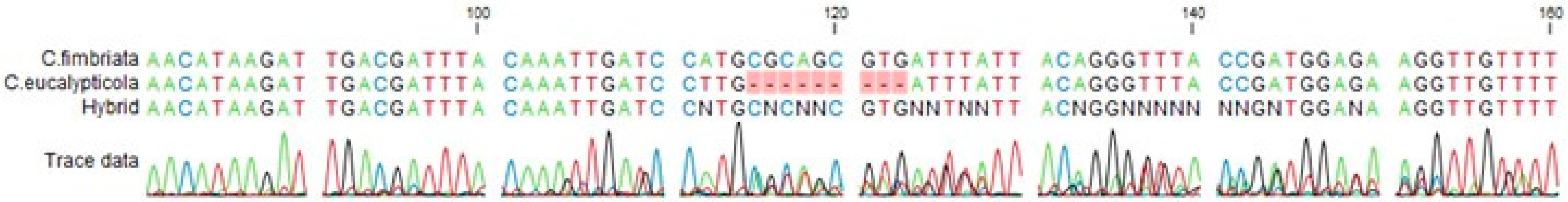

Figure S3 Electropherograms from sequencing the amplicons using the mitochondrial sequencing primer. Amplicons generated with mitochondrial primer set 1 yielded distinctly different sequencing profiles for the two parents and their hybrid progeny. The sequence of *C. fimbriata* has a 9 bp insertion whereas this sequence is absent in *C. eucalypticola* . There is a clear presence of two sequencing profiles in the region of the 9 bp insert in the hybrid progeny of this cross, indicative of the presence of both parent’s mitochondrial DNA.

Table S1. RFLP results showing the parental origin of individual spore drops for three self-fertile interspecific crosses generated during the first round of crosses.

Round 1	*C. fimbriata* x *C. eucalypticola*			*C. fimbriata* x *C. manginecans*			*C. eucalypticola* x *C. manginecans*	
Spore drop	Marker 2 origin	Marker 3 origin	Spore drop	Marker 1 origin	Marker 3 origin	Spore drop	Marker 1 origin	Marker 2 origin
fe 1 A1	F*	F	fm 1 A1	M	M	em 1 A1	M	M
fe 1 A2	E	E	fm 1 A2	F	F	em 1 A2	E	E
fe 1 A	H	H	fm 1 A	F	F	em 1 A	M	M
fe 1 B	H	H	fm 1 B	F	F	em 1 B	E	E
fe 1 C	H	H	fm 1 C	F	F	em 1 C	E	E
fe 1 D	H	H	fm 1 D	F	H	em 1 D	E	E
fe 1 E	H	H	fm 1 E	F	F	em 1 E	E	E
fe 2 A	-	E	fm 2 A	F	F	em 2 A	E	-
fe 2 B	H	F	fm 2 B	F	F	em 2 B	-	E
fe 2 C	H	E	fm 2 C	F	F	em 2 C	E	E
fe 2 D	H	E	fm 2 D	F	F	em 2 D	E	E
fe 2 E	F	F	fm 2 E	F	F	em 2 E	E	E
fe 3 A	H	H	fm 3 A	F	F	em 3 A	E	E
fe 3 B	F	F	fm 3 B	F	F	em 3 B	E	E
fe 3 C	F	F	fm 3 C	F	F	em 3 C	E	E
fe 3 D	F	F	fm 3 D	F	F	em 3 D	E	E
fe 3 E	H	H	fm 3 E	F	F	em 3 E	E	E
fe 4 A	E	E	fm 4 A	F	F	em 4 A	E	E
fe 4 B	H	F	fm 4 B	F	F	em 4 B	E	E
fe 4 C	E	E	fm 4 C	F	F	em 4 C	E	E
fe 4 D	-	E	fm 4 D	-	-	em 4 D	E	E
fe 4 E	E	E	fm 4 E	F	F	em 4 E	E	E
fe 5 A	H	H	fm 5 A	F	F	em 5 A	E	E
fe 5 B	H	H	fm 5 B	F	F	em 5 B	E	E
fe 5 C	H	H	fm 5 C	M	M	em 5 C	E	E
fe 5 D	H	H	fm 5 D	F	H	em 5 D	E	E
fe 5 E	H	H	fm 5 E	F	H	em 5 E	E	E

* F indicates the banding pattern produced by *C. fimbriata*, E indicates the banding pattern for *C. eucalypticola* , M indicates the banding pattern for *C. manginecans*, and H stands for hybridization, meaning that the banding pattern observed was a combination of two individual parents. A1 and A2 were positive controls.

Table S2. RFLP results showing the parental origin of individual spore drops for three self-fertile interspecific crosses generated during the second round of crosses.

Round 2	*C. fimbriata x C. eucalypticola*		*C. fimbriata x C. manginecans*			*C. eucalypticola x C. manginecans*		
Spore drop	Marker 2 origin	Marker 3 origin	Spore drop	Marker 1 origin	Marker 3 origin	Spore drop	Marker 1 origin	Marker 2 origin
fe 1 A1	F	F	fm 1 A1	F	F	em 1 A1	M	M
fe 1 A2	E	E	fm 1 A2	M	M	em 1 A2	E	E
fe 1 A	E	E	fm 1 A	F	F	em 1 A	M	M
fe 1 B	H	H	fm 1 B	-	F	em 1 B	M	M
fe 1 C	E	E	fm 1 C	F	F	em 1 C	M	M
fe 1 D	H	H	fm 1 D	F	F	em 1 D	M	M
fe 1 E	E	E	fm 1 E	F	F	em 1 E	M	M
fe 2 A	E	E	fm 2 A	F	F	em 2 A	M	M
fe 2 B	H	E	fm 2 B	F	F	em 2 B	E	-
fe 2 C	E	E	fm 2 C	F	F	em 2 C	E	E
fe 2 D	E	E	fm 2 D	F	F	em 2 D	E	E
fe 2 E	H	E	fm 2 E	F	F	em 2 E	E	-
fe 3 A	H	E	fm 3 A	F	F	em 3 A	E	E
fe 3 B	E	E	fm 3 B	H	F	em 3 B	E	E
fe 3 C	H	H	fm 3 C	H	F	em 3 C	E	E
fe 3 D	E	E	fm 3 D	H	F	em 3 D	E	E
fe 3 E	E	E	fm 3 E	F	-	em 3 E	E	-
fe 4 A	E	E	fm 4 A	F	F	em 4 A	E	E
fe 4 B	H	E	fm 4 B	F	F	em 4 B	E	E
fe 4 C	E	E	fm 4 C	F	F	em 4 C	E	E
fe 4 D	H	E	fm 4 D	F	F	em 4 D	E	E
fe 4 E	E	E	fm 4 E	H	F	em 4 E	E	E
fe 5 A	H	E	fm 5 A	F	F	em 5 A	E	E
fe 5 B	E	E	fm 5 B	H	F	em 5 B	E	E
fe 5 C	E	E	fm 5 C	F	F	em 5 C	E	E
fe 5 D	H	E	fm 5 D	F	F	em 5 D	E	E
fe 5 E	E	E	fm 5 E	F	F	em 5 E	E	E
